# Cameras or Divers? How Baited Remote Underwater Videos and ‘Long Swims’ Underwater Visual Census Complement Each Other on Coral Reefs

**DOI:** 10.1002/ece3.73533

**Published:** 2026-04-22

**Authors:** Kennedy Osuka‐Edeye, Melita Samoilys, Bryce D. Stewart, Colin J. McClean, Peter Musembi, Saleh Yahya

**Affiliations:** ^1^ Department of Earth, Ocean and Ecological Sciences University of Liverpool Liverpool UK; ^2^ CORDIO East Africa Mombasa Kenya; ^3^ Marine Biological Association Plymouth UK; ^4^ University of Plymouth Plymouth UK; ^5^ Department of Environment and Geography University of York York UK; ^6^ Wildlife Conservation Society‐ Kenya Marine Program Mombasa Kenya; ^7^ Institute of Marine Science University of Dar es Salaam Dar es Salaam Tanzania

**Keywords:** coral reef fishes, fish community, fish monitoring, trade‐offs, videography

## Abstract

Conventional methods for surveying coral reefs, such as underwater visual census (UVC), often underestimate large‐bodied, mobile, or cryptic fish species due to limitations in diver mobility and visual detection. To address these limitations, rapid timed ‘Long Swim’ (LS) UVC protocols have been developed to better capture large predator assemblages, yet their performance relative to baited remote underwater videos (BRUV) remains poorly evaluated in topographically complex oceanic reef environments. This study provides the first site‐specific comparison of BRUVS and LS‐UVC protocols for predatory reef fish from nine selected families on the reefs of Pemba Island, Tanzania. To ensure robustness, we implemented post hoc spatial filtering, a multivariate sensitivity analysis excluding bait‐attracted Carangidae, and site‐level depth‐ and effort‐matched bootstrap resampling. Community composition differed significantly between methods (PERMANOVA *p* = 0.001), a pattern that persisted even when bait‐attracted carangids were excluded from the analysis. After standardising for effort and depth, BRUVs detected higher species richness and a greater number of unique species, particularly mobile and deep‐dwelling taxa. In contrast, LS‐UVC excelled at detecting residential reef‐associated taxa but recorded fewer unique species. Jaccard similarity between methods remained consistently low (< 0.2), indicating that each method samples distinct ecological components of the predator community. These findings highlight that the primary value of integrating BRUVs and LS‐UVC lies in capturing complementary components of the predator assemblage rather than strictly increasing overall richness estimates. Their combined use provides a more comprehensive and ecologically meaningful assessment, essential for managing predatory reef fish communities in complex habitats and depths.

## Graphical abstract

This study compares baited remote underwater videos (BRUV) and a rapid timed long swim (LS) UVC method to evaluate their ability in assessing relative abundance and diversity of large predatory reef fish from nine selected families. BRUVs were more effective in detecting mobile and bait‐attracted taxa such as Carangidae and recorded up to 28 unique predatory species. LS‐UVC was good for surveying demersal reef‐associated taxa such as Lethrinidae and recorded two unique species. These findings highlight the complementary value of both methods, supporting their combined use for a more comprehensive and ecologically meaningful assessment of predatory reef fish communities.

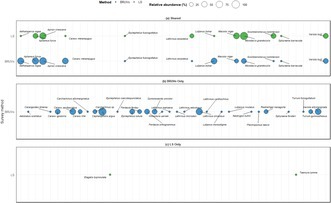

## 1 Introduction

Large predatory reef fish constitute a critical protein resource for millions of coastal inhabitants around the world (Sadovy de Mitcheson et al. 2020; Béné et al. 2015). These species also play a significant ecological role in regulating populations and community structure of their prey (Stewart and Jones 2001; Boaden and Kingsford 2015). Owing to their substantial body size, these fish are preferentially targeted by fishermen. However, the intrinsic characteristics of the life history of these species, namely, slow growth rates and delayed sexual maturation, make them especially susceptible to overexploitation and subsequent declines in abundance (Worm et al. 2013; Samoilys et al. 2025). The resulting scarcity, particularly in regions subjected to intense fishing pressure, requires the development and application of robust methodologies to accurately quantify their populations.

Traditional reef survey approaches, such as the standard underwater visual census (UVC) conducted by divers on SCUBA, are susceptible to underestimation errors (Samoilys and Carlos 2000). Over several decades, a substantial body of literature has addressed these challenges, developing UVC protocols specifically for medium‐ to large‐bodied commercially important species and rare taxa that typically occur at low densities (Kulbicki and Sarramégna 1999; Rojo et al. 2021). These advancements include the use of distance sampling to refine density estimates and towed‐diver surveys for mesoscale assessments of large‐bodied reef fishes (Richards et al. 2011). Part of the underestimation is evident for species that have large home ranges or high mobility, exhibit cryptic behaviour in response to divers, which can affect their detectability during visual surveys. Highly mobile predators and species with large home ranges are often underrepresented in diver‐based surveys but may be more readily detected by remote camera systems (e.g., Cappo et al. 2004; Leujak and Ormond 2007; Langlois et al. 2018). Similarly, cryptic or sheltering species may be overlooked during visual censuses (Stewart and Beukers 2000) while some fishes may avoid or be disturbed by the presence of divers, potentially leading to underestimation of their abundance in diver‐based surveys (Kulbicki and Sarramégna 1999; Emslie et al. 2018). Consequently, a rapid timed long swim (LS) UVC survey method was designed to more accurately detect large‐bodied and often predatory reef fishes, as well as pelagic taxa that associate with reefs (Samoilys et al. 2025). However, UVC is impractical for surveying habitats located at depths beyond safe SCUBA limits, or in areas that are logistically challenging for divers to access (Harvey et al. 2001). In response to these limitations, baited remote underwater videos (BRUVs) have increasingly been adopted for surveying large‐bodied species. BRUVs offer the dual advantages of sampling across a broad range of depths and attracting large often overfished species (Harvey et al. 2012; White et al. 2013), in addition to providing permanent video records and information on habitat that may be re‐examined as necessary. However, BRUVs are not without challenges, including long video processing times and the inability to assign the values of the indices obtained from counts to a defined area, and therefore to calculate density (Langlois et al. 2018). Given that each survey methodology is associated with inherent biases, an integrated approach is increasingly advocated to achieve a comprehensive assessment of reef fish communities. However, the relative merits of BRUVs and rapid timed LS UVC have only rarely been directly compared at the same place and time (but see Cheal et al. 2021).

An integrated methodological framework enables the survey of extensive and well‐defined reef areas within shallower waters via UVC, thus mitigating the limited field of view challenge inherent to BRUVs, while simultaneously facilitating the assessment of fish communities across a gradient of shallow to moderately deep habitats through BRUVs. This dual approach allows for the comparison of in situ visual records collected from UVC and BRUV video data (Cappo et al. 2004). Synthesis of data from both BRUVs and UVC is expected to enhance existing monitoring efforts by providing a finer resolution of the structure of the reef fish community (Schramm et al. 2020). Despite inter‐method biases, studies conducted on the Great Barrier Reef have empirically demonstrated that the integration of BRUVs and UVC yields a more complete perspective of reef fish assemblages, thus facilitating more informed decision‐making by resource managers (Cheal et al. 2021). Consequently, a similar integration of survey methods is hypothesised to be beneficial for other diverse coral reef systems, such as those surrounding the oceanic island of Pemba in the Western Indian Ocean.

The coral reefs that surround Pemba Island in Tanzania are renowned for steep drop‐offs and strong currents, habitats known to attract larger predatory reef fish (Grimsditch et al. 2009; Gudka et al. 2024; Osuka et al. 2021). However, they are increasingly threatened by local fishing practices, including the historic and deleterious impacts of dynamite fishing, which have resulted in severe degradation, particularly in unprotected areas. To date, only a limited number of investigations have examined the reefs around Pemba Island (Horrill 1992; Horrill et al. 1994; Daniels et al. 2004; Grimsditch et al. 2009; Jones et al. 2019; Gudka et al. 2024), with a predominant research focus on Misali Island in the southern region. Consequently, there is an urgent need to identify practical and cost‐effective monitoring protocols to support the conservation and management of this ecologically significant area.

Given the focus on documenting large and potentially rare species, we used an extended UVC timed LS measuring an area of seabed of approximately 3000 m^2^, which has been identified as more suitable for monitoring large predatory reef fish than conventional transect dimensions (e.g., 50 m × 5 m) (Mapstone and Ayling 1998; Samoilys et al. 2025). In this study, we assess the performance of BRUVs and a rapid timed LS visual census (LS‐UVC) in terms of estimating abundance and accurately representing the taxonomic composition of large predatory fish assemblages, as well as capturing their species richness. We hypothesise that BRUVs will more effectively detect pelagic and highly mobile predatory fishes known to respond to bait plumes (hereafter ‘bait‐attracted taxa’) including families such as Carangidae, Scombridae, and Sphyraenidae. In contrast, LS‐UVC is expected to be more effective at quantifying residential reef‐associated species that are less responsive to bait and remain closely associated with reef habitat. To evaluate the relative performance and potential complementarity of these methods, we evaluated the similarities and differences in fish assemblages recorded during BRUV deployments (Osuka et al. 2022) and LS‐UVC transects (Samoilys et al. 2025) in the same reef sites on Pemba Island.

## 2 Materials and Methods

### 2.1 Study Area

**FIGURE 1:**
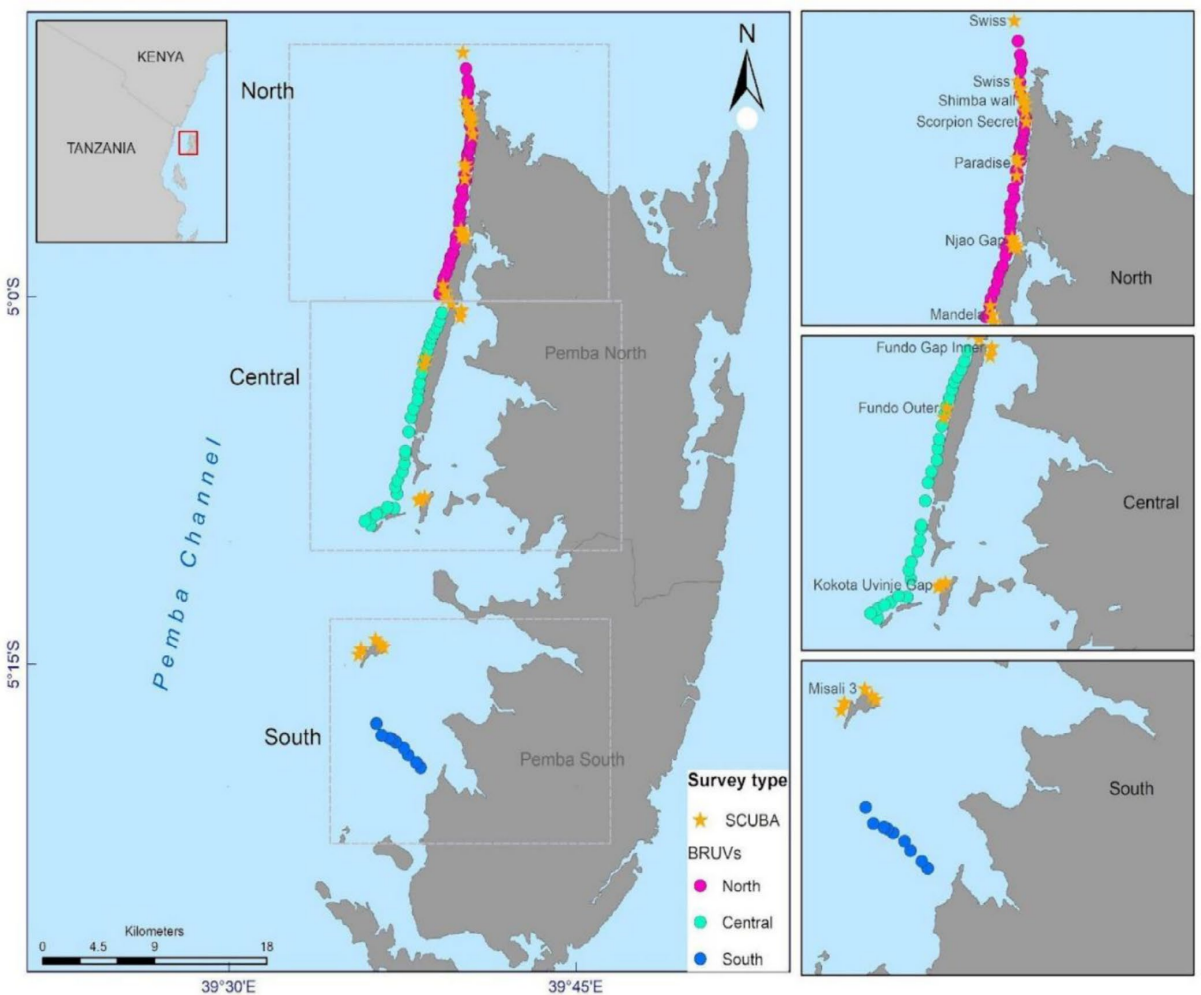
Map of the study stations where baited remote underwater videos (BRUVs) were deployed (filled circles), and long swim underwater visual census on SCUBA (filled stars) were conducted off Pemba Island.

The BRUV and LS‐UVC surveys were conducted along the outer reefs of the western margins of Pemba Island. Sampling sites were selected based on previous surveys within the Greater Pemba Channel, particularly those conducted using SCUBA (Grimsditch et al. 2009) and autonomous underwater vehicles (Osuka et al. 2021). These sites were chosen to represent diverse reef habitats used by predatory reef fish (Figure 1).

### 2.2 Description of the Surveys

A total of 61 mono‐BRUV deployments (Osuka et al. 2022) and 23 LS‐UVC surveys (Samoilys et al. 2025) were conducted at seven reef sites from northern to southern Pemba Island in November 2019 (Table 1). Sampling effort was allocated to ensure that the relative number of replicates for each method at each site represented similar proportions of the total sampling effort per method, thereby facilitating comparability between BRUV and LS‐UVC surveys while minimising site‐level sampling bias.

**TABLE 1:** Number of replicates and depth range per site for baited remote underwater videos (BRUV) and rapid timed ‘long swims’ underwater visual census (LS‐UVC) conducted on coral reefs of Pemba Island.

Sector	Site	BRUVs		LS‐UVC	
		No. of replicates	Depth range (m)	No. of replicates	Depth range (m)
South	Misali—Vikunguni	5	10–39	2	8–21
Central	Kokota—Uvinje	7	6–40	2	2–18
Central	Fundo	15	8–43	6	4–21
North	Mandela	5	15–47	2	7–20
North	Njao	11	7–40	4	7–26
North	Paradise	8	10–45	2	5–19
North	Shimba—Scorpion Secret	10	7–40	3	4–20
	Total	61	6–47	23	2–26

#### 2.2.1 BRUVS Surveys

Each sampling event involved the deployment of a mono‐BRUV unit. The unit consisted of a GoPro Hero4 Silver video camera, configured with a medium field of view (FOV), and mounted on a stainless steel frame. A 1.65 m conduit pipe was placed within the camera's FOV and used to support a meshed bait bag. To minimise bias in fish attraction and detection rates, the bait type, quantity, and preparation method were standardised across all BRUVs deployments. Each bait bag was filled with approximately 1 kg of oily fish, mainly from the Scombridae and Carangidae families, which were chopped into small pieces to enhance scent dispersion and attraction efficiency (Dorman et al. 2012). Although precise species‐level consistency in bait type can be difficult to maintain in remote island settings where bait availability depends on daily catches, the bait was standardised by functional type, quantity and preparation method across all samples. The bait bag was securely attached to the conduit pipe to ensure consistent placement in all deployments.

For deployment, the BRUV unit was tethered using a rope tied to the top of the frame and connected to a surface buoy, allowing accurate positioning, detection and retrieval. Each deployment lasted a minimum of 1 h (Table 2), with replicate units spaced approximately 500 m apart along the reef to prevent overlap in bait plumes and fields of attraction (Harvey et al. 2007). A minimum of five replicate deployments was carried out per site, under the assumption that each unit sampled a comparable area. Abundance estimates were recorded as the maximum number of individuals observed in a single frame (MaxN) (Langlois et al. 2018).

**TABLE 2:** List of variables and their measurements for baited remote underwater videos (BRUVs) and rapid timed ‘long swims’ underwater visual census (LS‐UVC).

Variable	BRUVs	LS
Time	~1 h video recording	10 min swims on SCUBA
Replicates per reef	At least 5	At least 2
Distance between replicates/stations	~500 m	~470 m
Reef area	NA	~150 m × 20 m (3000 m^2^)
Resolution	Medium FOV [1080]	NA
Measurement of abundance	Maximum number (MaxN) seen in a video footage	Absolute counts (N)

Abbreviations: FOV, field of view; NA, not applicable.

#### 2.2.2 LS‐UVC Surveys

At each selected reef site, two replicate LS‐UVC surveys were conducted by an observer (MS) throughout, spaced an average of 470 m apart and at depths ranging from 2 to 26 m (Table 2). Each survey consisted of 10‐min timed swim parallel to the reef crest, covering the reef depth gradient to a maximum of 30 m and an area of approximately 150 m × 20 m (3000 m^2^) (Samoilys et al. 2025). Abundance estimates were recorded as total counts of individuals observed during LS‐UVC (Samoilys and Carlos 2000). Unlike traditional belt transects, the LS‐UVC approach allows observers to track mobile taxa across heterogeneous reef habitats, increasing encounter probability. It is worth noting that while the sampling area for the LS‐UVC protocol used in this study is visually estimated, we acknowledge that new methods now exist for more accurately measuring and mapping census objects without significant additional equipment, such as GPS‐tracked roaming transects (Lynch et al. 2015; Irigoyen et al. 2018, 2025).

### 2.3 Target Taxa

Predatory reef fishes were identified at the species level in both survey methods. The target species were selected based on their ecological importance, exploitation in artisanal fisheries and size‐related rarity (Samoilys and Carlos 2000; Kawaka et al. 2016). Predominantly large, fast‐swimming epipelagic species, including members of Carangidae, Carcharhinidae, Dasyatilidae, Myliobatidae, Scombridae and Sphyraenidae (Appendix 1), were surveyed (Mapstone and Ayling 1998; White et al. 2013; Osuka et al. 2022; Samoilys et al. 2025). For families commonly evaluated in UVC surveys, the demersal Lethrinidae, Lutjanidae and Epinephelinidae, only individuals with a maximum total length of > 55 cm were included in the analysis to ensure comparability with other studies of large predator species (Samoilys et al. 2025; Heupel et al. 2013; Paxton et al. 2020).

### 2.4 Data Processing and Analysis

#### 2.4.1 Influence of Distance Between Methods

A generalised additive model (GAM) was used to assess the effect of distance between the LS‐UVC and BRUV stations on relative abundance and species richness (Cheal et al. 2021). Although no significant effect on relative abundance was found, species richness decreased significantly at BRUV stations located more than 4 km from the nearest LS‐UVC station (Appendix 2). Consequently, BRUV deployment locations beyond this distance were excluded from further analysis.

#### 2.4.2 Method Comparisons

To evaluate the ability of each method to capture large predatory reef fish communities, we compared the frequency of occurrence of common species and families in BRUV and LS‐UVC surveys. The mean abundance was first calculated for each species and family in all replicates (Table 1). To account for differing abundance metrics (MaxN for BRUVs and absolute counts for LS‐UVC), abundances were expressed as relative contributions within each survey method. We divided the mean abundance of each species or family at a site by the total mean abundance across all taxa within that specific method. This standardised approach allowed for a robust comparison of detection frequency and assemblage structure across sites despite differing sampling units.

To explore the complementarity of BRUVs and LS‐UVC, we examined the detection of rare species separately, highlighting those uniquely recorded by one method but not the other. A Venn diagram was employed to illustrate the distribution of the number of species unique to BRUVs and LS and those shared between the two methods. This approach allowed us to assess how combining methods may offer a more complete picture of the predator assemblage. Paired *t*‐tests were performed to compare species abundance estimates between methods, after checking for parametric testing of normality and homogeneity of variances.

#### 2.4.3 Depth and Effort Matching

To account for unequal sampling effort and differences in depth distributions between the survey types, we applied site‐level depth‐ and effort‐matched bootstrap resampling. Within each site, the survey with greater replication (number of stations) was resampled with replacement to match the number of stations in the lower effort survey. Resampling was weighted by similarity to the depth distribution of the reference survey using a Gaussian kernel, ensuring that the resampled deployments reflected the LS depth domain. This resulted in only five sites being used in the depth and effort matching analysis (Paradise, Njao, Mandela, Kokota Uvinje and Fundo). For each bootstrap iteration (*n* = 999), species detections were converted to presence–absence and pooled within survey type at the site level. A combined curve was also produced using data from both BRUVs and LS‐UVC to evaluate the potential cumulative benefit of integrating the two methods for broader predator community characterisation. Assemblage similarity between BRUVs and LS‐UVC was then quantified using the Jaccard similarity index. This procedure generated a distribution of Jaccard similarity values for each site, from which mean similarity and 95% confidence intervals were estimated.

#### 2.4.4 Sensitivity Analysis

To test whether bait‐attracted taxa influenced differences between survey methods, we conducted a sensitivity analysis focusing on Carangidae, a family known to respond strongly to bait in BRUV deployments (e.g., Jones et al. 2020). Community composition was analysed using PERMANOVA (*adonis2*, vegan package Oksanen et al. 2007; R Core Team 2021) based on Bray–Curtis dissimilarities of Wisconsin‐standardised abundance data, with survey method as the explanatory factor and 999 permutations. To quantify the potential influence of disproportionate bait attraction, the analysis was repeated after excluding Carangidae from the community matrix. Comparing results from the full dataset and the reduced dataset allowed us to assess whether compositional differences between methods were driven primarily by this bait‐attracted family.

## 3 Results

### 3.1 Species Frequency of Occurrence

**FIGURE 2:**
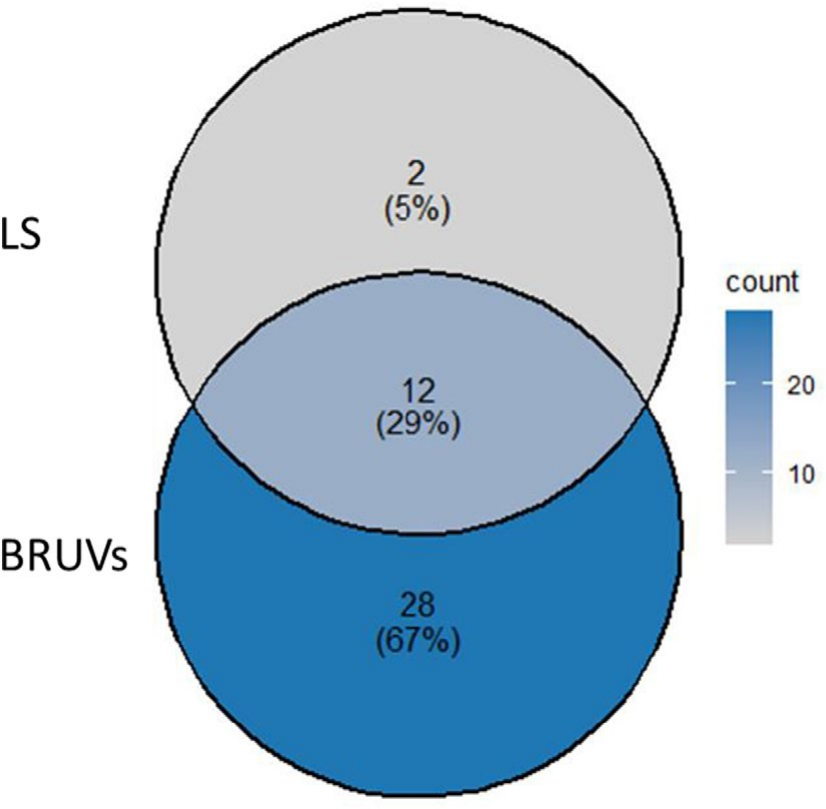
A Venn diagram showing the number and percentage distribution of species occurring in surveys conducted using baited remote underwater videos (BRUVs) and long swims underwater visual census LS‐UVC.

A total of 42 species were encountered in both methods. Of these, 95.2% and 33.3% were observed in BRUVs and LS‐UVC, respectively. Twelve species were common in both methods, while 28 and two species were unique to BRUVs and LS‐UVC, respectively (Figure 2).

**FIGURE 3:**
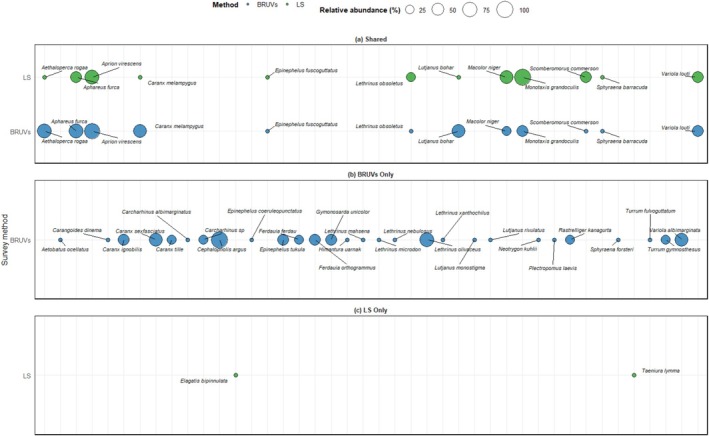
Frequency of occurrence of fish species (a) common to baited remote underwater videos (BRUVs) and ‘long swims’ (LS) underwater visual census; (b) species unique to BRUVs; and (c) species unique to LS underwater visual census.

The frequency of occurrence of common species revealed the presence of *Monotaxis grandoculis* in all sites sampled by LS‐UVC compared with only 42.9% in the sites surveyed by BRUV (Figure 3a). The occurrence of *Aprion virescens* showed the least difference between LS‐UVC and BRUV, being present in more than 70% of the sites. Other species that frequently occurred in BRUVs were *Aphareus furca* and *Aethaloperca rogaa*, both occurring in 71.4% of the sites compared with < 43.0% of the sites surveyed by LS‐UVC.

Two distinct species unique to BRUVs were *Cephalopholis argus* and *Lethrinus olivaceus*, which were recorded in more than 70% of sites. The majority (54%) of the unique species recorded in the BRUVs were sampled in 14.3% of the sites (Figure 3b). Unique species sampled by LS‐UVC were *Taeniura lymma* and *Elagatis bipinnulata*, each found in 14.3% of the sites (Figure 3c).

### 3.2 Families and Species Abundance

**FIGURE 4:**
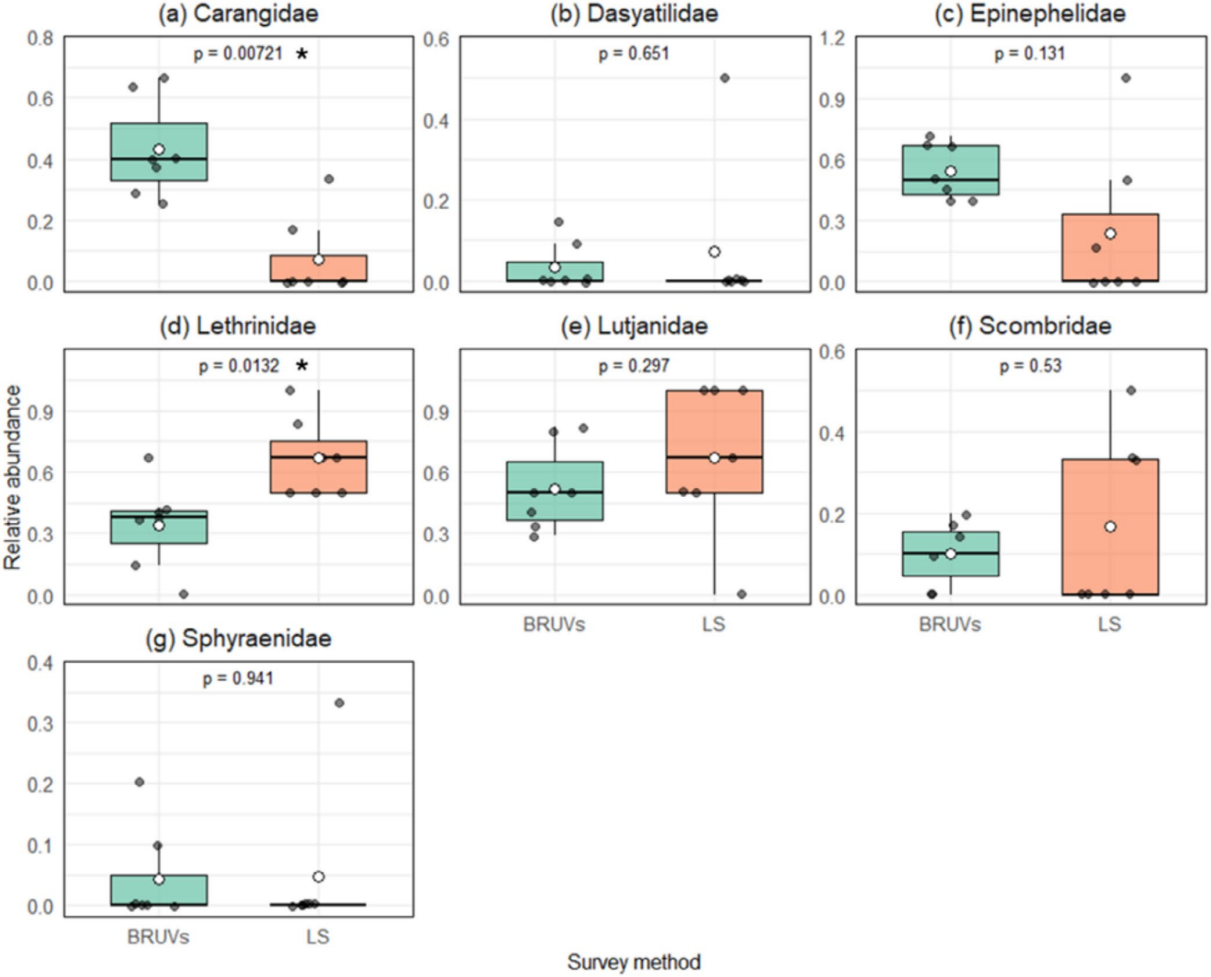
Median (interquartile range) and mean (white point) relative abundance for fish families shared by baited remote underwater video (BRUV) shaded in green and long swims (LS) underwater visual census surveys shaded in orange, conducted on Pemba Island, Tanzania. The *p*‐values of the paired *t*‐test are included with those significant shown in asterisks.

**FIGURE 5:**
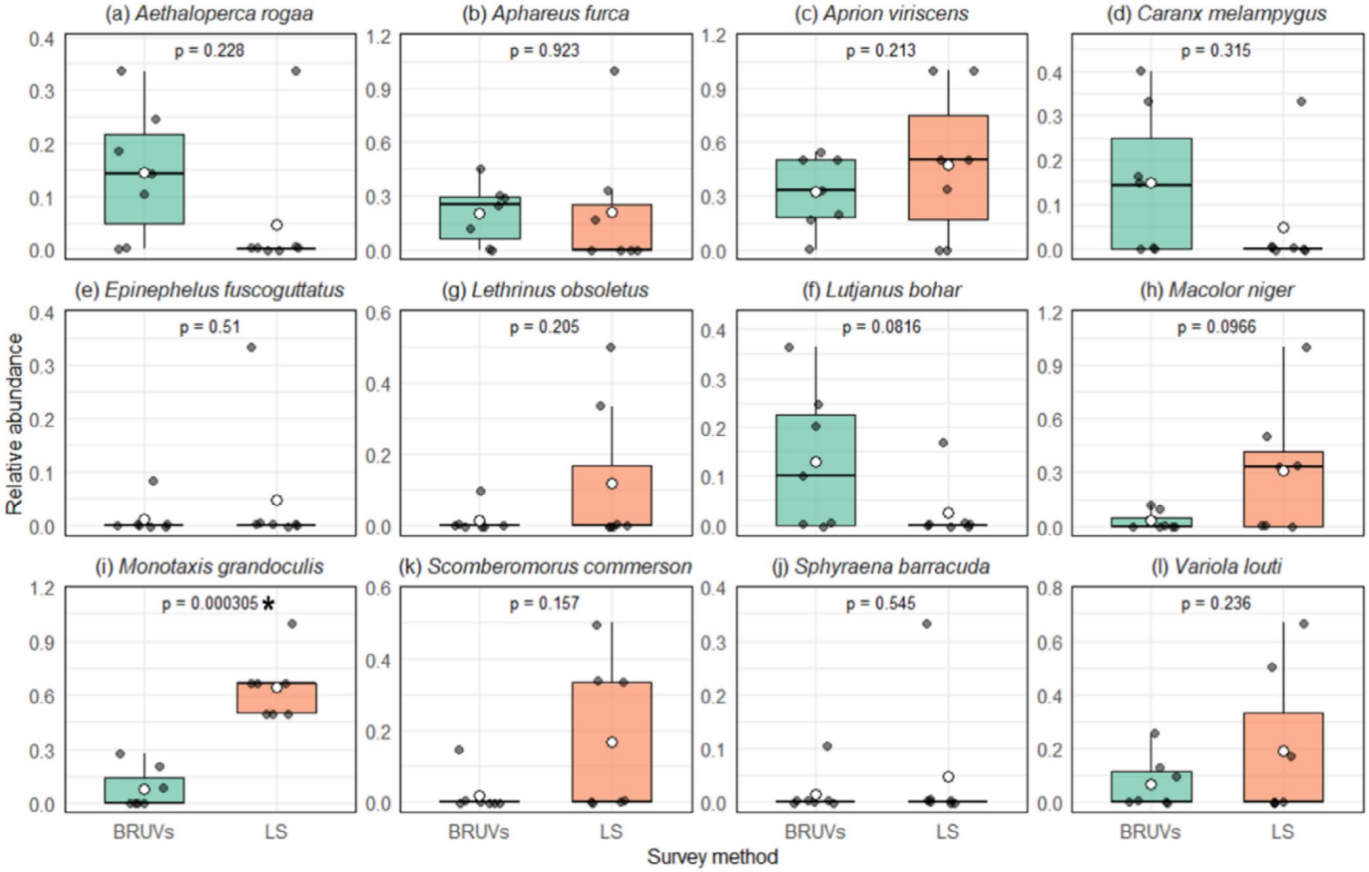
Median (interquartile range) and mean (white point) relative abundance for species shared by baited remote underwater video (BRUV) shaded in green and long swims (LS) underwater visual census surveys shaded in orange, conducted in Pemba Island, Tanzania. The *p*‐values of paired *t*‐test are included with those significant shown in asterisks.

Of the nine families surveyed, only Carangidae and Lethrinidae showed significant differences between the two survey methods (Figure 4a,d). The relative abundance of Carangidae was higher in BRUVs than in LS‐UVC (*t*‐test *t* = 3.169; *p* < 0.001) while an opposite pattern was observed for Lethrinidae (*t*‐test *t* = −2.725; *p* = 0.013). Other families did not show clear significant differences between survey methods. The paired *t*‐tests revealed that LS‐UVC and BRUV were only different in recording the abundance of one of the 12 shared species (Figure 5i). Lethrinid *Monotaxis grandoculis* showed higher abundance in LS‐UVC than in BRUV (*t*‐test, *t* = 4.800; *p* < 0.001). While these family‐specific differences, particularly in Carangidae, suggest a potential methodological bias, we conducted a multivariate sensitivity analysis to determine if these trends fundamentally altered the perceived community structure.

### 3.3 Community Composition and Sensitivity Analysis

Community composition differed significantly between survey methods when all families were included (PERMANOVA: *F* = 5.59, *R* ^2^ = 0.075, *p* = 0.001), indicating that BRUVs and LS‐UVC detected distinct family‐level assemblages. Excluding Carangidae did not change this pattern. Survey method remained highly significant (*F* = 5.74, *R* ^2^ = 0.087, *p* = 0.001), and the proportion of variance explained increased slightly. This indicates that differences between survey methods were not driven by highly bait‐attracted Carangidae, and that methodological complementarity persists after accounting for potential bait attraction effects. Beyond taxonomic composition, the robustness of these findings was further tested by standardising sampling effort and depth strata through bootstrap resampling to ensure that observed complementarity was not an artefact of unequal coverage.

### 3.4 Depth and Effort Matching

**FIGURE 6:**
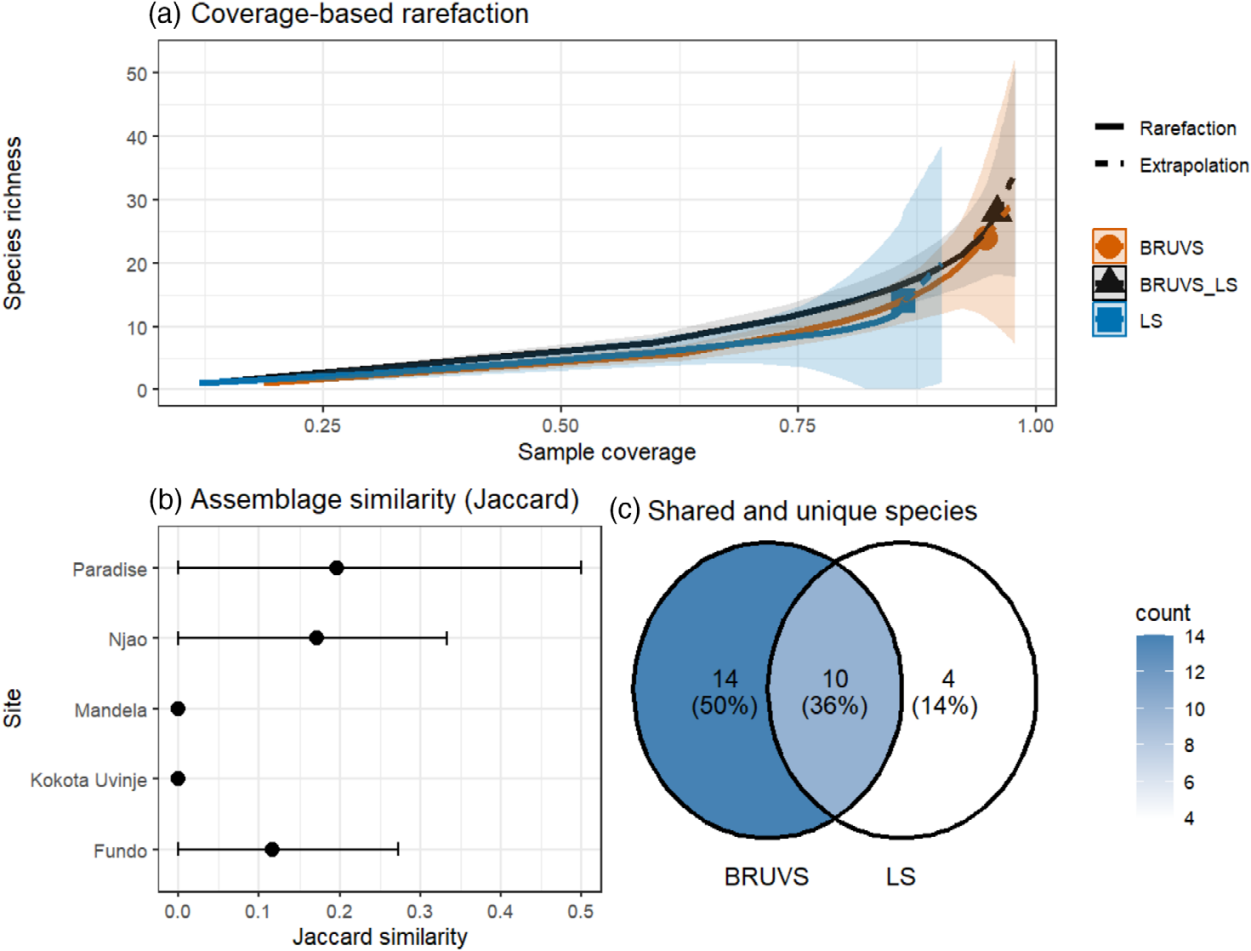
Comparison of reef fish assemblages detected by BRUVs and LS surveys. (a) Coverage‐based rarefaction curves showing species richness as a function of sample coverage. Solid lines represent interpolation and dashed lines extrapolation, with shaded areas showing 95% confidence intervals. (b) Jaccard similarity indices (mean ±95% CI) derived from 999 bootstrap resamples matching BRUVs sampling effort to LS effort within the LS depth domain. (c) Venn diagram illustrating the number of species detected uniquely by each method and those shared between BRUVS and LS‐UVC surveys.

BRUVs detected higher species richness than LS‐UVC across comparable levels of sample coverage, with extrapolated curves indicating a continued increase in richness for BRUVs relative to LS‐UVC (Figure 6a). Assemblage similarity between methods was generally low across sites, with mean Jaccard indices typically < 0.2 and overlapping confidence intervals (Figure 6b). At some sites (e.g., Kokota Uvinje and Mandela), no shared species were detected between methods across all bootstrap iterations, resulting in similarity estimates of zero. Where overlap was observed (e.g., Fundo, Paradise and Njao), the number of shared species remained low, averaging approximately one species per site.

Confidence intervals around similarity estimates frequently included zero, indicating that shared detections were consistently rare. Overall, 14 species (50%) were detected exclusively by BRUVs and four species (14%) exclusively by LS‐UVC, while 10 species (36%) were shared between both survey methods (Figure 6C). BRUVs generally identified a greater number of unique species (mean 2.2–7.8 species per site), whereas LS‐UVC surveys recorded between 0.8 and 4.0 unique species per site.

## 4 Discussion

### 4.1 Complementarity and Geographic Novelty

This study provides the first site‐specific comparison of BRUVs and the LS‐UVC protocol for reef fish assemblages in Pemba Island, Tanzania. While the general complementarity of baited video and visual census is relatively well documented (e.g., Cheal et al. 2021), previous studies have largely focused on fixed‐length and fixed‐width transects rather than roaming survey approaches. For example, Cheal et al. (2021) compared BRUVs with conventional UVC transects of 1 m width for small and cryptic species and 5 m width for larger, mobile taxa, but did not evaluate large‐scale roaming visual surveys. By contrast, LS‐UVC protocol represents a roaming census approach that allows observers to track mobile species over larger spatial extents, potentially providing a more appropriate methodological comparison with BRUV deployments (Samoilys et al. 2025). Importantly, the study demonstrates the value of integrating these methods lies not in strictly increasing species richness estimates, but in capturing complementary components of the predator assemblage. The greater number of unique species and the low Jaccard similarity between methods highlights their complementary strengths, indicating that they capture different ecological ‘niches’ within the predator community, particularly across depth ranges and mobility gradients.

BRUV and LS‐UVC are complementary survey methods to evaluate reef fish communities, each excelling under different conditions and for different species types. This is particularly evident in the contrasting abundance patterns of Carangidae and Lethrinidae. Both methods detected mobile taxa, indicating that mobility is not the main factor influencing detectability. Although the broader coverage of LS‐UVC might be expected to improve the detection of rarer, patchily distributed species, the results only partially support this pattern. Such differences underscore the value of combining both methods to avoid underestimating key and rare taxonomic groups. Coral reef fish population surveys face inherent biases tied to methodological approaches and resulting abundance estimations, necessitating the combination of methods for enhanced accuracy and informed management decisions (Andradi‐Brown et al. 2016). Among the various assessment methods, BRUV and LS‐UVC have emerged as prominent techniques, each providing unique insights into the structure of the fish community of large‐bodied species (Cheal et al. 2021; Choat and Pears 2003; Goetze et al. 2021; Langlois et al. 2018; Robbins et al. 2006; Samoilys et al. 2025).

In habitats where monitoring needs to extend to depths beyond 20–30 m, BRUVs are generally essential due to logistical and safety constraints on diving. In contrast, LS‐UVC surveys remain critical in shallow, high‐complexity reef environments where bait plumes may be less effective and divers can capture nuanced habitat–species associations. Understanding these differences is critical for designing effective monitoring programmes and informing marine conservation strategies (Emslie et al. 2018; Cheal et al. 2021).

### 4.2 Taxa‐Occurrence Patterns

BRUVs were more effective in detecting mobile, transient, and bait‐attracted species such as Carangidae, Carcharhinidae and Aetobatidae and recorded 28 unique predatory species not observed in LS‐UVC surveys (see Appendix 1). Even after controlling for depth and effort, BRUVs were associated with detecting 14 unique species. Carangid species tend to move in schools, respond rapidly to bait plumes, and often inhabit a broader depth range, making them well suited for detection via stationary baited video systems (Stewart and Jones 2001; Hixon 2015). According to a recent study, the high abundance of carangid species in BRUVs is associated with sandy areas that are widespread in the deeper marine areas of Pemba Island (Osuka et al. 2022). These findings support the integration of BRUVs into the Global Coral Reef Monitoring Network (GCRMN) protocols, where monitoring of mobile (and often important for fisheries) taxa attracted by bait, such as Carangidae, is a priority, ensuring consistency, detectability, and comparability between regions (GCRMN 2021).

LS‐UVC surveys are designed primarily to detect mobile pelagic and epipelagic species, such as sharks, barracuda and scombrids, which are typically underrepresented in standard UVC methods (Samoilys et al. 2025). However, in Pemba Island, LS‐UVC outperformed BRUVs in detecting certain taxa beyond their expected scope. LS‐UVC recorded two unique species ( *Elagatis bipinnulata* and *Taeniura lymma* ) alongside demersal species closely associated with reef structure, such as members of the Lethrinidae family and *Monotaxis grandoculis* . LS‐UVCs cover larger spatial areas than standard UVCs and BRUVs and allow divers to detect mobile species, increasing chances of encountering fast‐swimming pelagics like *Elagatis bipinnulata* and cryptic, reef‐associated rays like *Taeniura lymma* . The demersal *Monotaxis grandoculis* often forms large aggregations of more than 50 individuals and exhibits limited movement (Lieske and Myers 1994), resulting in patchy distribution. Lethrinids, in general, are associated with sandy and coral reef habitats, where they feed on relatively sedentary prey (Kulbicki et al. 2005). These results show that LS‐UVC surveys may have broader utility and are well suited to shallow, structurally complex habitats where fine‐scale ecological interactions and structure‐associated species can be more effectively observed in situ. Despite a shorter survey duration (10 min), LS‐UVCs produced higher relative abundances of these taxa, likely due to the divers' ability to traverse reef habitats where these species reside (Kulbicki et al. 2005; Wilson et al. 2018; Samoilys et al. 2025). These patterns reinforce behavioural and ecological traits that are relevant for the detection of fish taxa, with BRUVs excelling with roving or bait‐attracted taxa, while LS‐UVCs favour demersal species that may not respond to bait or may be underrepresented within the camera's FOV (Colton and Swearer 2010; Cheal et al. 2021).

Both BRUVs and LS‐UVC demonstrated comparable effectiveness in detecting several key reef‐associated fish families, including Dasyatidae, Epinephelidae (e.g., *Aethaloperca rogaa* , *Epinephelus fuscoguttatus* , *Variola louti* ), Lutjanidae (e.g., *Aphareus furca* , *Aprion virescens* , *Lutjanus bohar* , *Macolor niger* ), Scombridae (e.g., *Scomberomorus commerson* ) and Sphyraenidae (e.g., *Sphyraena barracuda* ). Statistical comparisons did not reveal significant differences in occurrence rates for these families and species, indicating a convergence in the capabilities of both methods. This similarity likely reflects a shared sensitivity to mobile predatory species, mid‐ to large‐bodied, which are more readily detected due to their size, swimming behaviour, and spatial range. Similar findings have been reported in other reef systems, where BRUV and UVC methods yielded convergent estimates of species richness and abundance, particularly for large mobile taxa (Langlois et al. 2010), underscoring the complementary value of these methods in reef fish monitoring.

### 4.3 Accessibility to Depth and Habitat

BRUVs offer the critical advantage of accessing a wider depth range. While LS‐UVCs are typically restricted to less than 30 m due to SCUBA limitations, BRUVs can be deployed at much greater depths, enabling researchers to access deeper reef habitats that can act as refugia from fishing pressure or climate impacts (Osuka et al. 2022). BRUVs would be well suited for depth‐stratified monitoring due to their ability to operate across a wide range of depths (Whitmarsh et al. 2017). This makes them useful for detecting species that inhabit or traverse broader depth gradients, such as members of the family Scombridae, including *Gymnosarda unicolor* and *Rastrelliger kanagurta* . BRUVs also proved effective in recording a high diversity of trevally species (Carangidae), capturing unique species such as *Carangoides dinema* , *Ferdauia ferdau*, *Turrum fulvoguttatum*, *Turrum gymnostethus*, *Ferdauia orthogrammus*, *Caranx ignobilis* , *Caranx sexfasciatus* and *Caranx tille* . It is likely that the presence of bait increases the likelihood of species appearing in the BRUV footage. This may be due to the bait plume effectively dispersing across the area, attracting pelagic and roving demersal species. Furthermore, the long soak time in BRUV increases the likelihood of detecting elusive or shy species, such as *Aethaloperca rogaa* , which can enter the FOV only after extended periods (Harvey et al. 2012; Espinoza et al. 2014). This long soak time is also critical to increasing the likelihood of recording large mobile species (Currey‐Randall et al. 2020).

### 4.4 Methodological Biases

Survey methods introduce different biases that need to be acknowledged and/or corrected for. For instance, the size of the area surveyed, and the use of bait, can influence abundance estimates when utilising BRUVs (Cheal and Thompson 1997; Harvey et al. 2007). Regarding LS‐UVC surveys, potential bias would emerge from avoidance effects of divers by fishes, leading to poor detectability and underestimation of targeted species (Kulbicki et al. 2010; Gray et al. 2016; Bradley et al. 2017; Heenan et al. 2020). In the specific context of Pemba Island's steep drop‐offs and strong currents, these biases manifest clearly: the high mobility of carangids in the deep Pemba Channel likely enhances their detection via the BRUV bait plumes, whereas the structural complexity of the shallow reef crests allows LS‐UVC to more accurately capture residential, reef‐associated lethrinids that may avoid stationary cameras or ignore bait. Both methods present trade‐offs in logistics and data handling (Table 3). BRUVs provide a permanent video record, allowing species identifications to be verified or re‐analysed (Harvey et al. 2012). However, they are labour‐intensive in post‐processing, as each 1‐h deployment requires at least equivalent review time, and often much more (Langlois et al. 2018). LS‐UVCs offer faster data turnaround, with observations recorded in real time, but are heavily relying on diver expertise for accurate identification. The sampling area in LS‐UVC used here is visually estimated at ~3000 m^2^. While this provides standardised unit for biomass estimates (Samoilys and Carlos 2000; Heenan et al. 2017), it lacks the high precision of ‘Tracked Roaming Transects’ or distance sampling. These established methods may be superior for monitoring rare or low‐density species as they allow for the accurate mapping and measurement of census objects, increasing the efficiency of UVCs (Irigoyen et al. 2018; Kulbicki and Sarramégna 1999; Beck et al. 2014). BRUVs rely on an undefined FOV, which may vary due to reef features or water clarity (Colton and Swearer 2010). Cost‐wise, BRUVs avoid ongoing expenses related to diving logistics and insurance but require a significant upfront investment in specialised equipment and software, along with expert capacity for video analysis. On the contrary, LS‐UVCs can be more cost‐effective in areas with existing dive infrastructure, although they incur continuous costs for diver insurance, gear maintenance, and adherence to safety SCUBA protocols (Heenan et al. 2017; Osuka, unpublished data). This makes BRUVs a more scalable option for national monitoring programmes, especially when resources are limited. Taken together, the findings indicate that between the two methods, BRUVS are often the better choice. However, the preferred option is to use a combination of BRUVs and LS‐UVC.

**TABLE 3:** Summary of the trade‐offs between baited remote underwater videos (BRUVs) and long swim underwater visual census (LS‐UVC).

Criteria	BRUVs	LS‐UVC
Sampling area	Cannot be estimated but relies on the field of view (FOV) set on the camera	Based on timed swims in a visually estimated area (e.g., 3000 m^2^)
Depth limitations	As a remote video, depth of deployment is not constrained except by availability of suitable cameras and lights	LS‐UVC on SCUBA is logistically difficult to achieve > 30 m depth due to SCUBA limitations
Permanent record	Offers a permanent video record that can be reviewed by other scientists at other times	Collects data in situ, meaning full reliance on diver expertise, although can be supplemented with diver photography and video
Cost	While BRUVs eliminate the recurring costs of diver insurance and life‐support logistics, their high initial investment in equipment, limited availability in some regions, and the need for specialised video analysis software and expertise make them a costly option to establish and maintain	LS‐UVC surveys can be more cost‐effective in regions with established diving capacity, but they incur ongoing expenses related to diver insurance, gear upkeep, and safety protocols, which can be significant over time
Labour	Takes fewer days to survey an extensive reef area. However, it is labour‐intensive due to the high number of replicates, each requiring the same amount of time to analyse the videos as the recording time. Video processing happens back in the lab	Associated with less processing time, because species‐level data are collected in situ ready for rapid transcription to database in the field

### 4.5 Integration of Methods for Comprehensive Monitoring

The differences between methods point to a clear recommendation: Integration is essential to achieve a comprehensive understanding of reef fish communities across the full depth and habitat gradient. Using both approaches together leverages their complementary strengths, capturing the full spectrum of taxa from shallow, demersal species to deep‐dwelling, mobile predators. This integrated approach is particularly important for spanning reef zones, including mesophotic ecosystems, which are beyond the reach of standard UVC but are increasingly recognised for their biodiversity and conservation value. Although some taxa may elude one method or the other due to behavioural traits or habitat use (e.g., field of view limitations or bait avoidance/lack of response), combining survey types improves detection probabilities, especially for rare or cryptic species (Kulbicki et al. 2005; Colton and Swearer 2010). Tailoring an integrated design to habitat structure, species ecology, and depth profiles will produce more robust, ecologically meaningful data for monitoring and management (Harvey et al. 2012; Espinoza et al. 2014).

### 4.6 Implications for Conservation and Future Research

The integration of BRUVs and LS‐UVCs in monitoring programmes provides a more robust basis for conservation planning and implementation, including the design and monitoring of marine protected areas (MPAs). Together, they can reveal critical habitats for predators and areas where certain key species aggregate during vulnerable life stages and species richness hotspots. Coverage‐based rarefaction curves at matched effort show that combined methods do not significantly yield higher species richness than BRUVs alone. This demonstrates that integrating these methods primarily serves to capture unique, complementary components of the predator assemblage rather than strictly boosting overall richness estimates.

Future studies should move beyond simple richness comparisons to employ detection‐aware modelling approaches (e.g., occupancy models) that explicitly separate effort effects from detection probability (Staudhammer et al. 2018). Furthermore, adopting stereo‐BRUVs (Langlois et al. 2018) and GPS‐tracked roaming UVC would improve biomass estimation and spatial precision respectively.

The study provides data from two reef fish population census methods that could be highly valuable to inform conservation and management decisions, including spatial closures and restrictions on fishing gear. Although each has limitations, their strengths are distinct and complementary. Used strategically, based on the target species, habitat type, and logistic constraints, they provide a powerful toolkit for reef fish monitoring across depth gradients and ecological contexts. In settings where a single method is insufficient, their combination offers a more comprehensive, cost‐effective, and ecologically sound approach to assessing and managing predatory reef fish communities.

## Author Contributions

**Kennedy Osuka‐Edeye:** conceptualization (lead), data curation (lead), formal analysis (lead), funding acquisition (equal), investigation (lead), methodology (equal), project administration (equal), visualization (lead), writing – original draft (lead), writing – review and editing (lead). **Melita Samoilys:** funding acquisition (equal), investigation (equal), methodology (equal), supervision (supporting), writing – review and editing (equal). **Bryce D. Stewart:** investigation (supporting), methodology (supporting), resources (supporting), supervision (equal), writing – review and editing (equal). **Colin J. McClean:** investigation (supporting), methodology (supporting), resources (supporting), supervision (equal), writing – review and editing (equal). **Peter Musembi:** investigation (supporting), methodology (supporting), writing – review and editing (equal). **Saleh Yahya:** data curation (supporting), investigation (supporting), methodology (supporting), writing – review and editing (supporting).

## Funding

This work was supported by UK Global Challenges Research Fund, NE/P021050/1.

## Conflicts of Interest

The authors declare no conflicts of interest.

## Data Availability

The R scripts used to generate the figures in this study are provided. Additionally, presence–absence data for the families and species assessed using BRUV and UVC‐LS methods are included in the appendix.

## Appendix 1 Species Recorded During Baited Remote Underwater Video (BRUV) and Timed Long Swim (LS) Surveys Around Pemba Island, Tanzania. Presence (1) and Absence (0) Indicate Detection by Each Method. Maximum Length Values (TL = Total Length, FL = Fork Length, WD = Width of Disc) Are Sourced From FishBase

**Table:**

Family	Species	Common name	Maximum length (cm)	Length type	BRUVs	LS‐UVC
Aetobatidae	*Aetobatus ocellatus*	Ocellated eagle ray	300.0	WD	1	0
Carangidae	*Carangoides dinema*	Shadow trevally	85.0	TL	1	0
	*Ferdauia ferdau*	Blue trevally	70.0	TL	1	0
	*Turrum fulvoguttatum*	Yellowspotted trevally	120.0	FL	1	0
	*Turrum gymnosthesus*	Bludger	90.0	TL	1	0
	*Ferdauia orthogrammus*	Island trevally	75.0	TL	1	0
	*Caranx ignobilis*	Giant trevally	170.0	TL	1	0
	*Caranx melampygus*	Bluefin trevally	117.0	FL	1	1
	*Caranx sexfasciatus*	Bigeye trevally	120.0	TL	1	0
	*Caranx tille*	Tille trevally	80.0	TL	1	0
	*Elagatis bipinnulata*	Rainbow runner	180.0	TL	0	1
Carcharhinidae	*Carcharhinus albimarginatus*	Silvertip shark	300.0	TL	1	0
	*Carcharhinus* sp				1	0
Dasyatidae	*Himantura uarnak*	Honeycomb stingray	200.0	WD	1	0
	*Neotrygon kuhlii*	Blue‐spotted stingray	70.0	TL	1	0
	*Taeniura lymma*	Ribbontail stingray	35.0	WD	0	1
Epinephelidae	*Aethaloperca rogaa*	Redmouth grouper	60.0	TL	1	1
	*Cephalopholis argus*	Peacock hind	60.0	TL	1	0
	*Epinephelus coeruleopunctatus*	White‐spotted grouper	76.0	TL	1	0
	*Epinephelus fuscoguttatus*	Brown‐marbled grouper	120.0	TL	1	1
	*Epinephelus tukula*	Potato grouper	200.0	TL	1	0
	*Plectropomus laevis*	Black‐saddled coral grouper	125.0	TL	1	0
	*Variola albimarginata*	White‐edged lyretail	65.0	TL	1	0
	*Variola louti*	Yellow‐edged lyretail	83.0	TL	1	1
Lethrinidae	*Lethrinus mahsena*	Sky emperor	65.0	TL	1	0
	*Lethrinus microdon*	Smalltooth emperor	80.0	TL	1	0
	*Lethrinus nebulosus*	Spangled emperor	87.0	TL	1	0
	*Lethrinus obsoletus*	Orange‐striped emperor	60.0	TL	1	1
	*Lethrinus olivaceus*	Longface emperor	100.0	TL	1	0
	*Lethrinus xanthochilus*	Yellowlip emperor	70.0	FL	1	0
	*Monotaxis grandoculis*	Humpnose big‐eye bream	60.0	TL	1	1
Lutjanidae	*Aphareus furca*	Small‐toothed jobfish	70.0	TL	1	1
	*Aprion virescens*	Green jobfish	112.0	TL	1	1
	*Lutjanus bohar*	Two‐spot red snapper	90.0	TL	1	1
	*Lutjanus monostigma*	One‐spot snapper	60.0	TL	1	0
	*Lutjanus rivulatus*	Blubberlip snapper	80.0	TL	1	0
	*Macolor niger*	Black and white snapper	75.0	TL	1	1
Scombridae	*Gymonosarda unicolor*	Dogtooth tuna	248.0	FL	1	0
	*Rastrelliger kanagurta*	Indian mackerel	42.1	TL	1	0
	*Scomberomorus commerson*	Narrow‐barred Spanish mackerel	240.0	FL	1	1
Sphyraenidae	*Sphyraena barracuda*	Great barracuda	200.0	TL	1	1
	*Sphyraena forsteri*	Bigeye barracuda	92.3	FL	1	0
