# Correction to: “Cameras or Divers? How Baited Remote Underwater Videos and ‘Long Swims’ Underwater Visual Census Complement Each Other on Coral Reefs”

**DOI:** 10.1002/ece3.73759

**Published:** 2026-05-29

Osuka‐Edeye, K., M. Samoilys, B. D. Stewart, C. J. McClean, P. Musembi, and S. Yahya. 2026. “Cameras or Divers? How Baited Remote Underwater Videos and ‘Long Swims’ Underwater Visual Census Complement Each Other on Coral Reefs.” *Ecology and Evolution* 16, no. 4: e73533. https://doi.org/10.1002/ece3.73533.

The city name in the affiliation for the last author, Saleh Yahya, was incorrect. The correct affiliation is:

Institute of Marine Sciences, University of Dar es Salaam, Zanzibar, Tanzania.

Additionally, the first sentence in the Data Availability Statement should read: “The R scripts used to generate the figures in this study are available upon request.”

We apologise for these errors.

